# Combination with Methotrexate and Cyclophosphamide Attenuated Maturation of Dendritic Cells: Inducing Treg Skewing and Th17 Suppression *In Vivo*

**DOI:** 10.1155/2013/238035

**Published:** 2013-09-30

**Authors:** Xiaoyang Yu, Caihong Wang, Jing Luo, Xiangcong Zhao, Lixing Wang, Xiaofeng Li

**Affiliations:** Department of Rheumatology, The Second Hospital of Shanxi Medical University, 382 Wu Yi Road, Taiyuan 030001, China

## Abstract

Immune disorder is considered the main pathogenesis of autoimmune diseases, such as rheumatoid arthritis (RA). The balance of the two special subsets of CD4^+^T cells, T helper cell 17 (Th17), and Regulator T cell (Treg) is the key factor of maintaining a normal immune response. Dendritic cells (DCs), which are the most powerful antigen-presenting cells, play an important role in regulating the balance of Th17 and Treg. The combination of disease modifying antirheumatic drugs (DMARDs) is an important strategy of RA therapy. In this study, we investigated the effect of MTX and CTX on DC maturation in ovalbumin (OVA) immunized mice. Th17 inflammatory response is stronger, while the level of DCs maturity is higher. In contrast, the immunosuppression of Treg is stronger. We found that MTX combined with CTX significantly inhibited the DCs maturity and downregulated the antigen presenting capacity of DCs. As a result, it reestablished a balance of Th17 and Treg. Our study adds a novel mechanism and therapeutic target of MTX combined with CTX for autoimmune disease treatment.

## 1. Introduction

 Autoimmune diseases are caused by immunomodulatory imbalance, which in turn disrupts the immune response. CD4^+^T cells are a key factor for the cause of autoimmune diseases, such as RA. There are two kinds of new CD4^+^T cell subsets, including T help cell 17 (Th17) and regulatory T cell (Treg). Th17 mainly secretes IL-17 and mediates inflammatory response. Treg, specific expression of Foxp3, maintains cell immune tolerance. Th17 and Treg are both differentiated from naïve T cells. Dendritic cells (DCs) are the most important antigen-presenting cells (APCs) in the upstream of immunomodulatory pathway. DCs can significantly stimulate the naïve T cells proliferation and activation, regulating the differentiation of naïve T cells to Th17 and Treg [1, 2]. Clinical studies have found that peripheral blood CD4^+^T lymphocyte apoptosis rate is lower than the control in RA patients, and the ratio of Th17/Treg increases. A newly research showed that the breaking of balance between Th17 and Treg and the changing of cytokine microenvironment are the main pathogenesis of many autoimmune diseases [3]. Meanwhile, Th17 and Treg imbalance is closely related to the regulation of DCs. 

Dendritic cells, which can activate the naïve T cells, are the most powerful professional antigen-presenting cells. The differentiation and development of DCs experiences immature and mature stages. Immature DCs (imDCs) are in the peripheral tissues, which are poor in stimulating mixed lymphocyte reaction (MLR) as they express low levels of MHC-II molecules and costimulatory molecules [4]. Due to dangerous/invading antigen or inflammatory factors, imDCs switch to the mature DCs (mDCs). mDCs, expressing high levels of MHC-II molecules, CD80, CD86, and chemokine receptors, are ideally situated to meet and initiate effector T cell activation, govern the type of T-cell response, and alter the immune response profile *in vivo* [5, 6].

For the treatment of rheumatoid arthritis, early combination of disease modifying antirheumatic drugs (DMARDs) has reached a consensus in the world. It has been confirmed that combination therapy with cell cycle specific drug methotrexate (MTX) and nonspecific drug cyclophosphamide (CTX) has a significant clinical effect [7]. MTX specifically delays the transition from G0/G1 to S phase. CTX is a bifunctional alkylating agent, which can damage cells in any phase. Clinical data showed that compared with the single drug, combination with MTX and CTX significantly inhibits cell proliferation [8]. The mechanism studies showed that MTX combined with CTX can reduce levels of inflammatory cytokines, that is, TNF-*α*  and IL-1 and downregulate the expression of p53 and cyclin D1 mRNA. We have found that MTX combined with CTX has a synergistic effect [9].

In our study, using ovalbumin (OVA) immunized mice, we analyzed the effects of combination with MTX and CTX on maturation of DCs. We found that maturation of DCs was blocked in MTX combined with CTX-treated OVA immunized mice. The antigen presenting capacity of DCs was inhibited by MTX combined with CTX, which in turn inhibited OVA specific T cell proliferation and regulated the balance of Th17 and Treg. This may be a novel mechanism and therapeutic target of MTX combined with CTX for autoimmune disease treatment.

## 2. Materials and Methods

### 2.1. Animals

C57BL/6J mice, male, 6–8 weeks old, were purchased from Beijing Vital River Laboratory Animal Technology Co. Ltd. Mice were maintained under pathogen-free conditions. All procedures were performed in accordance with the Animal Care and Committee guidelines.

### 2.2. OVA Immunization

Ovalbumin (Sigma) was dissolved in sterile saline (2 mg/mL), which was emulsified with an equal volume of complete Freund's adjuvant (CFA). Mice were immunized with ovalbumin emulsion (OVA, 100 ug/mouse) s.c. [10] and then divided into four groups, which were untreated group (OVA group) and treatment groups (MTX, CTX, and MTX combined with CTX group). MTX was purchased from Hengrui Medicine Co. Ltd. (Jiangsu, China), and CTX was purchased from Pude Pharmaceutical Co. Ltd. (Shanxi, China). MTX and CTX were dissolved in sterile saline and injected into mice, 3.03 mg·kg^−1^·w^−1^ and 80.88 mg·kg^−1^·3w^−1^ i.p., respectively, for 9 weeks. Sterile saline was used as the control. We do experiments on the day before the first treatment and on the third, the sixth, and the ninth weeks.

### 2.3. Bone Marrow-Derived DCs Generation

Bone marrow cells were collected from femur and tibia of mice and were cultured in fresh DC culture medium (complete RPMI 1640 medium with 20 ng/mL rmGM-CSF and 20 ng/mL rmIL-4 (PeproTech)). Cultures were incubated at 37°C in 5% CO_2_ for 7 days to obtain immature DCs. Immature DCs were induced with 10 ug/mL LPS (Sigma) for 48 h. Semisuspended cells and loose adherent cells were harvested, which were mature bone marrow DCs (BMDCs).

### 2.4. Antibodies and Flow Cytometry

Single cell suspensions were prepared and cells were stained with fluorochrome-labeled or biotin-conjugated antimouse monoclonal antibodies (mAbs): CD11c, CD40, CD80, CD86, and IA-IE. These cells were incubated for 30 minutes at 4°C with primary antibody or antibodies and washed twice with fluorescence-activated cell sorting (FACS) buffer (PBS/2% bovine serum albumin/0.1% azide). Flow cytometry was performed using FACS Calibur cytometer (BD Biosciences) and analyzed using CellQuest software (Becton Dickinson).

### 2.5. Purification of CD4^+^T Cells

The purification of CD4^+^T cells has been previously described. Fresh spleens were removed and prepared for single-cell suspensions. CD4^+^T cells were negatively isolated using magnetic bead separation. In brief, splenocytes were depleted of CD8^+^, B220^+^, CD16^+^, Gr-1^+^, and Ly76^+^ cells using biotin-labeled specific mAb (Miltenyi Biotec), antibiotin magnetic beads (Miltenyi Biotec), and an LD magnetic bead column (Miltenyi Biotec). The CD4^+^T cells were purified using CD4^+^T cell Isolation Kit (Miltenyi Biotec) according to the manufacturer's instructions. The purity of CD4^+^T cells and DCs was always greater than 95%.

### 2.6. Cell Proliferation Assay *In Vitro*

Purified DCs (2 × 10^4^ per well) from treated (MTX and CTX group) and untreated (OVA group) mice were cocultured with purified allogeneic CD4^+^T cells (1 × 10^5^ per well) derived from naïve mice with complete RPMI-1640 in triplicate wells of 96-well plates for 72 h. Cultures were incubated at 37°C in 5% CO_2_ and pulsed with [^3^H]-thymidine (100 ul per well, 3.7 × 10^4^ Bq/mL) (Shanghai Institute of Atomic Nucleus, Chinese Academy of Sciences, China) for 16 h before harvest. [^3^H]-thymidine incorporation was measured as count pulse per minute (cpm).

### 2.7. Flow Cytometric Analysis of Th17 and Treg Cells [11]

For analysis of Th17 cells, PBMCs were suspended at a final density of 0.5 × 10^7^ cells/mL in complete RPMI 1640 culture medium. Cultures were stimulated for 5 h with 30 ng/mL phorbol myristate acetate (PMA), 750 ng/mL ionomycin, and brefeldin A. Cells were washed in FACS solution and surface-labeled with CD4-APC. Following surface staining, cells were fixed and permeabilized using fixation/permeabilization reagent (Becton Dickinson) and then stained with IL-17-PE (Th17). For analysis of Treg cells, PBMCs were aliquoted into tubes without PMA and ionomycin stimulation and surface labeled with CD4-FITC and CD25-PE followed by fixation, permeabilization, and intracellular staining with FoxP3-FITC. Labeled cells were washed and analyzed with a FACSCalibur flow cytometer (Bec ton-Dickinson) using the CellQuest software (Becton-Dickinson). In each case, staining was compared with that of the appropriately labeled isotype control antibody.

### 2.8. Statistical Analysis

All values shown in graphs represent the mean (±SEM). The difference among groups was determined by ANOVA analysis or Kruskal-Wallis *H* test, and comparison between two groups was analyzed by the *t*-test. *P* value less than 0.05 was considered statistically significant.

## 3. Results

### 3.1. Surface Antigen's Expression of BMDC on Different Time of Administration

The surface antigen's expression of BMDC from mice of treatment group (MTX, CTX, and MTX combined with CTX group), untreated group (OVA group), and controls were determined by flow cytometry. Before the first treatment (0 week), we detected the expression levels of BMDC surface molecules. The levels of CD40, CD80, CD86, and MHC-II in OVA challenged mice all exhibited a significant increase compared with those of control mice (*P* < 0.05) (Table 1). No significant difference of CD11c was observed between the two groups (*P* = 0.072). DC maturation is a critical process in immune mediated inflammatory reaction. The DC maturation of OVA-immunized mice showed a significant increase, which also confirmed that OVA had induced the inflammatory response.

MTX and CTX attenuated DC maturation. With the time of treatment, the expression levels of DCs surface molecules in every treatment group all decreased. Compared with 0 week and 3rd week, respectively, although the DC maturation of 3rd and 6th weeks showed a little decrease in every treatment group, no significant difference was observed (*P* > 0.05). The DC maturation was significantly decreased on 9th week (Tables 3, 4, and 5). We have not found significant difference among different time of administration in OVA group (*P* > 0.05) (Table 2).

### 3.2. Comparison of Dynamic Changes about DC Surface Antigen's Expression in Each Group

**Figure 1:**
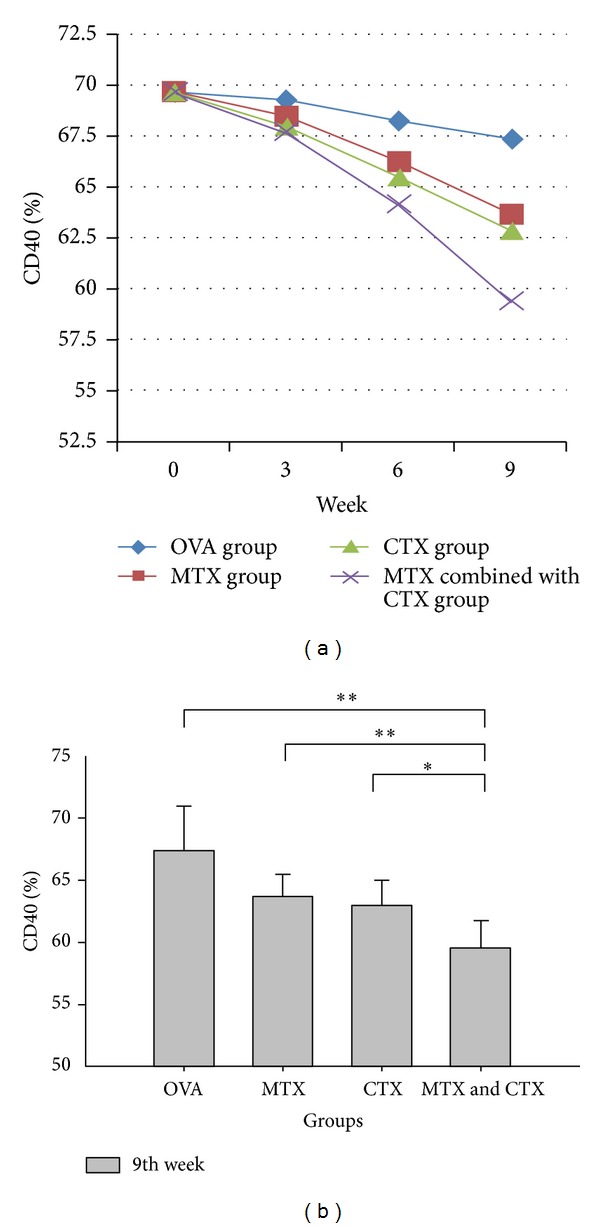
(a) Dynamic changes of CD40 expression in each group. (b) Comparison of CD40 expression in each group on 9th week. Data represented one of at least three independent experiments with 5 mice per group (*P < 0.05, **P < 0.01).

**Figure 2:**
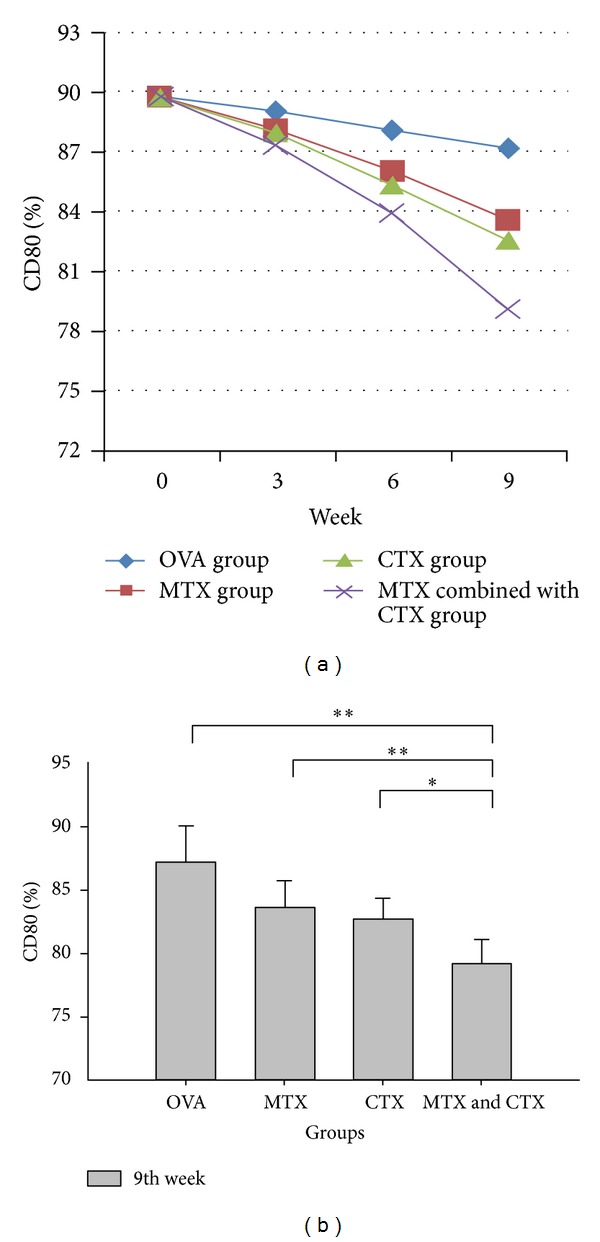
(a) Dynamic changes of CD80 expression in each group. (b) Comparison of CD80 expression in each group on 9th week. Data represented one of at least three independent experiments with 5 mice per group (*P < 0.05, **P < 0.01).

**Figure 3:**
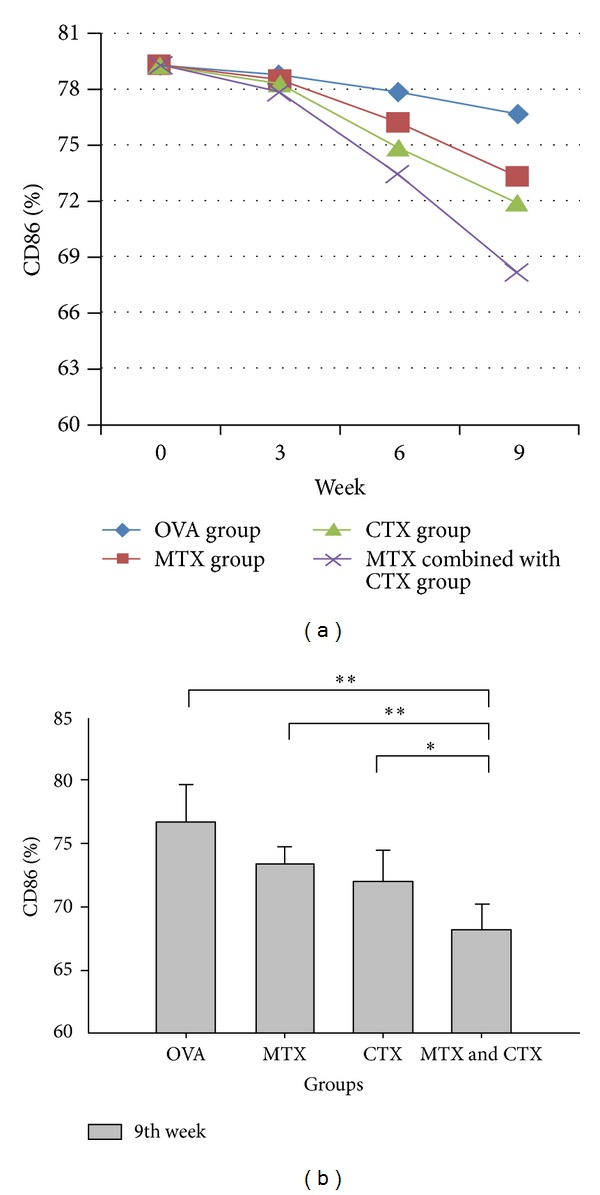
(a) Dynamic changes of CD86 expression in each group. (b) Comparison of CD86 expression in each group on 9th week. Data represented one of at least three independent experiments with 5 mice per group (*P < 0.05, **P < 0.01).

**Figure 4:**
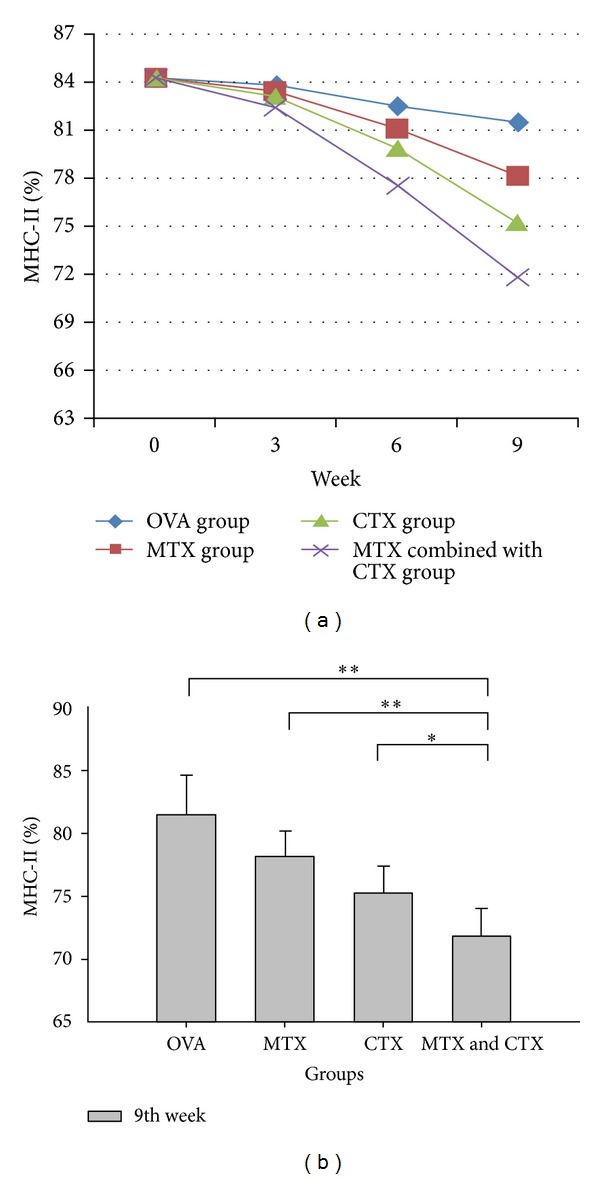
(a) Dynamic changes of MHC-II expression in each group. (b) Comparison of MHC-IIexpression in each group on 9th week. Data represented one of at least three independent experiments with 5 mice per group (*P < 0.05, **P < 0.01).

**Figure 5:**
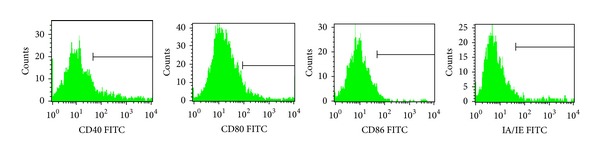
FACS analysis of BMDC surface antigen's expression in MTX combined with CTX group on 9th week. CD40 59.4%, CD80 79.0%, CD86 66.1%, and MHC-II 69.6%. Data represented one of at least three independent experiments with 5 mice.

 The expression levels of DC surface antigen (CD40, CD80, CD86, and MHC-II) showed a downward trend over time in the treatment group (Figures 1(a), 2(a), 3(a), and 4(a)). Compared with the single drug groups and OVA group, MTX combined with CTX group exhibited the most significant decrease on 9th week (the values of *P* were all less than 0.05). We showed the flow cytometry results of BMDC surface antigen's expression in MTX combined with CTX group on 9th week (Figure 5). As shown in Table 6, single drug groups (MTX or CTX group) expressed lower levels of DC surface antigen than those of control on 9th week (*P* < 0.05). There is no significant difference between the two single drug groups (*P* > 0.05). In analysis of the decline curve of DC surface antigen in each group, we found that the 3rd week may be a turning point of the curve. During the first 3 weeks, DC surface antigen exhibited a slow downward trend after being given DMARDs in the treatment group. Subsequently, the curve declined rapidly. It might be related to the drug onset time. Statistical analysis of dynamic changes about DC surface antigen's expression on different time showed that the expression levels' decrease is a gradual process.

### 3.3. MTX and CTX Suppressed Inflammatory Response in OVA Challenged Mice

**Figure 6:**
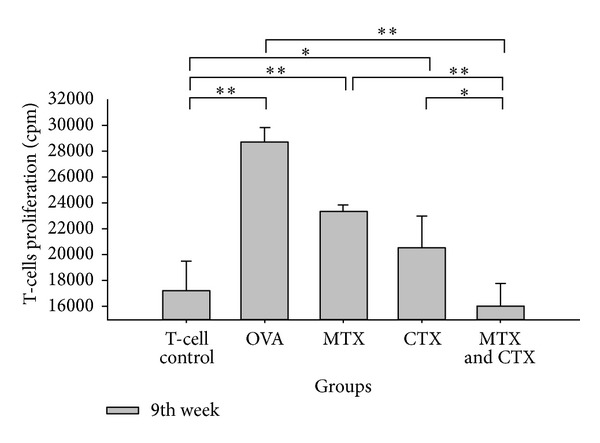
Stimulation index of mixed lymphocytes stimulated by BMDC on 9th week. Data were representative of 3 independent experiments (*P < 0.05, **P < 0.01).

To evaluate the immune suppression capacity of MTX and CTX *in vivo*, OVA-immunized mice were treated with MTX and CTX. A decrease of antigen-specific T cell proliferation was detected by [^3^H]-thymidine incorporation. Purified DCs from treated (MTX and CTX group) and untreated (OVA group) mice were cocultured with purified allogeneic CD4^+^T cells derived from naïve mice in 9th week. The results showed that proliferation of T cells from MTX and CTX-treated mice was significantly decreased (Figure 6). The T-cell proliferation of OVA group and single drug groups showed a significant increased compared with that of T cell control group (*P* < 0.05). No significant difference was observed between MTX combined CTX-treated mice and the control mice (*t* = 0.767, *P* = 0.461). Compared with OVA group, the proliferation of T cells from treatment groups was significantly decreased (*P* < 0.05). Combination group exhibited a significant decrease compared with MTX or CTX single used groups (*t* = 6.998, *P* = 0.012; *t* = 2.703, *P* = 0.035). The difference between single drug groups showed no significant difference (*P* > 0.05) (Table 7).

### 3.4. Correlation Analysis of DC Maturation and the Ratio of Th17/Treg *In Vivo*

**Figure 7:**
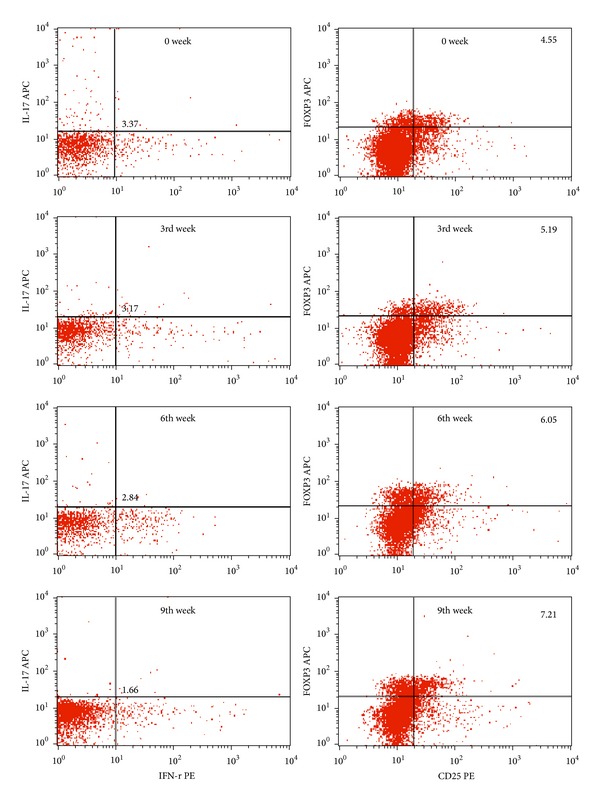
Flow cytometry image of Th17 (IL-17A^+^) and Treg (CD25^+^FOXP3^+^) from MTX combined with CTX-treated mice. Data represented one of at least three independent experiments with 5 mice.

**Figure 8:**
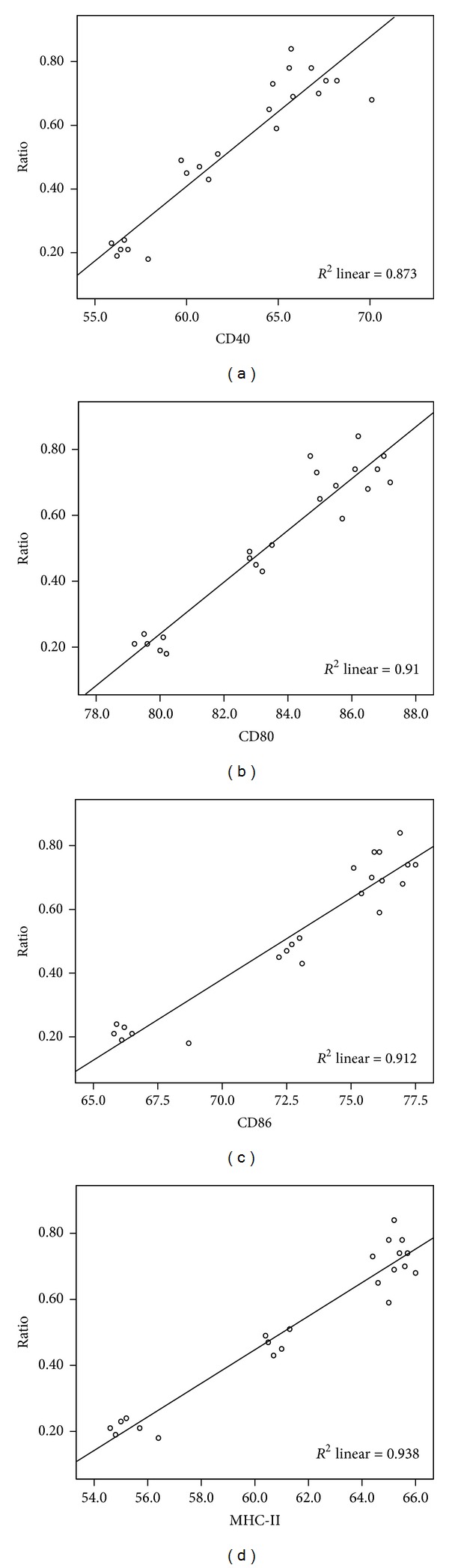
The correlation scatter plot of Th17/Treg ratio and DC surface antigen CD40 (*r* = 0.862, *P* < 0.001), CD80 (*r* = 0.855, *P* < 0.001), CD86 (*r* = 0.865, *P* < 0.001), and MHC-II (*r* = 0.860, *P* < 0.001).

A significant increase of inflammatory response could be detected in OVA challenged mice. The expression of DC surface antigen (CD40, CD80, CD86, and MHC-II) increased and induced antigen-specific T-cell proliferation. We found that the expression of antigen was downregulated and T cell proliferation decreased in MTX, CTX, and MTX combined with CTX-treated mice. To investigate whether the changes of DC surface antigen's expression was related to the ratio of Th17/Treg *in vivo*, we isolated splenocytes from MTX combined with CTX-treated mice on different time of administration. Flow cytometry analyzed the cell levels of Th17 and Treg (Figure 7). The results of Spearman rank correlation test showed that there was a positive correlation between the expression levels of DC surface molecules (CD40, CD80, CD86, and MHC-II) and the ratio of Th17/Treg. The correlation coefficients were, respectively, 0.862, 0.855, 0.865, and 0.860 (Figure 8).

The correlation was analyzed by Spearman rank correlation test. *P* < 0.05 was considered significant. The results exhibited a positive correlation between the expression levels of DC surface molecules (CD40, CD80, CD86, and MHC-II) and the ratios of Th17/Treg.

## 4. Discussion

 Autoimmune diseases are caused by immunomodulatory imbalance, which in turn disrupts the immune response. Rheumatoid arthritis (RA) is the most common chronic, systemic, inflammatory autoimmune disorder, affecting approximately 1% of the world's population [12]. Although the combination therapy of disease modifying antirheumatic drugs (DMARDs) and the use of biological agents have been made a lot of research progress, we have not reached the ultimate goal of long-term remission so as to reduce disability [13, 14]. It has been confirmed that combination therapy with cell cycle specific drug methotrexate (MTX) and nonspecific drug cyclophosphamide (CTX) has a significant clinical effect [7]. MTX specifically delays the transition from G0/G1 to S phase. CTX is a bifunctional alkylating agent, which can damage cells in any phase. Clinical data have showed that MTX combined with CTX has a synergistic effect, which can significantly inhibit the cell proliferation, reduce levels of inflammatory cytokines, that is, TNF-*α*  and IL-1, and downregulate the expression of p53 and cyclin D1 mRNA [8, 9]. 

 Immune disorder is considered the main pathogenesis of autoimmune diseases. CD4^+^T cells are a key factor for the cause of autoimmune diseases. There are two kinds of new CD4^+^T cell subsets, including T help cell 17 (Th17) and regulatory T cell (Treg). Th17 mainly secretes IL-17 and mediates inflammatory response. Treg, specific expression of Foxp3, maintains cell immune tolerance. The balance of Th17/Treg plays a great role in maintaining a normal immune response. Th17 and Treg are both differentiated from naïve T cells. Dendritic cells (DCs) are the most important antigen-presenting cells (APCs) in the upstream of immunomodulatory pathway. DCs can significantly stimulate the naïve T cells proliferation and activation, regulating the differentiation of naïve T cells to Th17 and Treg [1, 2]. Clinical studies have found that peripheral blood CD4^+^T lymphocyte apoptosis rate is lower than the control in RA patients, and the ratio of Th17/Treg increases. We have found that MTX combined with CTX could effectively reduce the Th17 proliferation in peripheral blood lymphocyte (PBL) of RA patients [7]. Then we attempt to investigate the main mechanism of MTX combined with CTX therapy from the point of view of DCs regulating Th17/Treg balance. 

In this study, we used ovalbumin (OVA) to challenge mice so as to make a model of immune disease. We investigated whether MTX combined with CTX suppressed the bone marrow-derived DCs (BMDC) maturation and then regulated the T cell proliferation and restored Th17/Treg immune balance. Actually, murine collagen-induced arthritis (CIA) has been a gold standard model of human rheumatoid arthritis (RA). However, an important limitation of the CIA model is that the collagen response and the disease are stimulated by exogenously injected collagen, whereas human RA is characterized by a spontaneous breach of selftolerance [15]. Thus, to be more applicable to the histologic and humoral features of human disease, we developed a model of ovalbumin- (OVA-) mediated polyarthritis, in which autoimmunity is spontaneous [15, 16]. OVA is a glycoprotein, which can be coupled with the small molecule hapten to become a complete antigen. It can induce autoimmune response. What is more, this study is based on the model of autoimmune disease, not just the RA model, avoiding the application limitation of the research results. 

Dendritic cell is a kind of cell with branch-like protrusions, which was first discovered from mouse spleen tissue by Ralph Steinman in 1973. Now, it has become one of the most active research fields in the immunology research. DCs can strongly stimulate T lymphocytes especially cytotoxic T lymphocytes (CTL) to produce the immune response. The differentiation and development of DCs experiences immature and mature stages. Immature DCs (imDCs) are in the peripheral tissues, which are poor in stimulating mixed lymphocyte reaction (MLR) as they express low levels of MHC-II molecules and costimulatory molecules [4]. Antigen presentation in the absence of costimulation can lead to impaired clonal expansion and T cell anergy [17]. Immature DCs are believed to induce T cell anergy or regulatory T cells (Treg) [18, 19]. When encountering a dangerous/invading antigen, imDCs migrate to the T cell area of secondary lymphoid organs, where imDCs switch to the mature DCs (mDCs) [10]. Mature DCs, expressing high levels of MHC-II molecules, CD80, CD86, and chemokine receptors, are ideally situated to meet and initiate effector T cell activation, govern the type of T-cell response, and alter the immune response profile *in vivo* [5, 6]. The ratio of mDC/imDC largely determines the differentiation and function of T cells and the immune response type, such as immune activation or tolerance [20].

The normal immune response needs DCs to provide two signals. The first signal is the antigen peptide compounds on the surface of mature DCs combining with T cell receptor (TCR) of naïve T cells. Then, the costimulatory molecules of DCs, such as CD40 and CD80/CD86, combine with CD40L and CD28 on the surface of T cells, respectively, which is the second signal. The two signals jointly initiate the acquired immune response, including naïve T cells clonal expansion and differentiation into effector T cells [21].

Our study found that the levels of CD40, CD80, CD86, and MHC-II in OVA challenged mice all exhibited a significant increase compared with those of control mice. It showed that OVA had activated the dendritic cells and initiated the immune response. The expression levels of DC surface antigen exhibited a downward trend over time in the treatment group. However, there was no significant difference during the first 3 weeks. The difference gradually emerged on 6th week, and the expression levels were significantly decreased on 9th week. That agreed with the function characteristics of the two DMARDs. The drugs had yet to pay off on 3rd week and on 6th week inhibition effect appeared gradually. Compared with MTX or CTX single used groups, DC surface antigen's expression showed a significant decrease in MTX combined with CTX-treated mice. The downward trend of CTX challenged mice was a little better than that of MTX single used group, but no significant difference was observed. Our study confirmed that DMARDs such as MTX and CTX could suppress the expression level of DC surface antigen. That is to say, they could suppress DC maturation and then reduce the antigen-presenting signal transmission. MTX combined with CTX exhibited the most significant suppression effect and reduced the acquired immune response. We considered that MTX or CTX could inhibit the stem cells function of the bone marrow, affect the differentiation of stem cells, and then change the expression level of BMDC surface antigen. Compared with MTX, CTX may have stronger inhibitory effect on BMDC.

Mixed lymphocyte reaction (MLR) needs the costimulatory molecules and MHC-II of DCs to provide the second signal so as to induce T cells activation. Our study showed that the changes of the DCs costimulatory molecules were consistent with the results of the MLR reactions. Compared with T cells control, the T cells proliferation of OVA immunized mice showed a significant increase. That means that due to a higher DC maturity, untreated OVA challenged mice showed a stronger antigen presenting reaction and immune response enhanced. In contrast, the T cells proliferation of combined treatment group exhibited no significant difference with the T cells control. Through the treatment of MTX combined with CTX, the T cells proliferation decreased and increased immune response tended to return to normal. That is because combined treatment significantly inhibited the expression levels of DCs costimulatory molecules and MHC-II, attenuated maturation of DCs, weakened antigen presenting reaction, and maintained immune tolerance. The T cells proliferation of single drug groups was somewhere between the two groups above, and no significant difference was observed. That also confirmed the superiority of MTX combined with CTX in immune regulation.

We found that there was a positive correlation between the expression levels of DC surface molecules and the ratios of Th17/Treg. That means that Th17 inflammatory response is stronger, while the level of DCs maturity is higher. In contrast, while MTX and CTX inhibited the DCs maturation, the immunosuppression of Treg is stronger. Th17 cells are pro-inflammatory cells characterized by the expression of IL-17A, IL-17F, IL-21, IL-22, IL-23R, and the transcription factors ROR-*γ*t and ROR-*α* [22, 23]. Treg, specific expression of Foxp3, produces TGF-*β*, IL-10, IL-35, and other inhibitory cytokines and inhibits inflammatory T cells proliferation and activation [24]. Dendritic cells, in the upstream of immunomodulatory pathway, regulate the balance of Th17 and Treg cells [25].

Mature DCs activate naïve T cells to produce a large number of effector T cells, for example, Th17, playing a critical role in immune-mediated inflammatory reaction [26]. Immature DCs promote the differentiation of Treg cells, mediating immune tolerance. Some cells in the mouse can coexpress Foxp3 and ROR-*γ*t [27, 28] and could represent an intermediate in this process or, alternatively, an intermediate cell in the differentiation of naïve T cells into Th17/Treg. The available data suggests that, although Foxp3 and ROR-*γ*t can be coexpressed in CD4^+^T cells, Foxp3 expression and IL-17 production are mutually exclusive [27, 29]. In DC maturation, Toll-like receptors (TLRs) play an instructive role through supplying the activation signal to induce the upregulation of MHC-II and costimulatory molecules on DCs [30, 31]. The activation of TLR7 and (or) TLR8, expressed on mature dendritic cells, can stimulate the Th17 cell differentiation [32]. Besides, mature DCs suppress Treg-mediated immune suppression by inducing IL-6 signal, which stimulates the differentiation of Th17 cells [33]. That raises the possibility that this could also contribute to the pathogenesis of autoimmune diseases [34] such as rheumatoid arthritis [35] or systemic lupus erythematosus [36]. 

Our study has several limitations. First, in this study, we made a preliminary discussion about DCs in MTX and CTX-treated mice regulating the balance of Th17/Treg. We focused on the balance of Th17/Treg, which is one of the most active research fields in the immunology research now, without involving the conventional T helper cells subsets, Th1 and Th2. However, it has been confirmed that DCs, in different maturity state, showed a different effect on T cell differentiation. Li et al. [37] have used atorvastatin to induce spleen-derived dendritic cells into tolerogenic DCs. Administration of these tolerogenic DCs to rats resulted in increased numbers of CD4^+^CD25^+^ T regulatory (Treg) cells and Foxp3 expression and shifted cytokine profile from Th1/Th17 to Th2 type cytokines. These tolerogenic DCs exerted their immunomodulatory effects mainly by decreased expression of CD86 and MHC class II on endogenous DCs. In addition, Dokić et al. [38] found that upon maturation DCs favored the production of Th2/Th17 cytokines by allogenic CD4^+^ lymphocytes in coculture, while immature DCs induced anergy, differentiation of suppressive CD4^+^CD25^high^CD39^+^ Treg-cell subsets, and increased production of TGF-*β* in the coculture. 

Second, IL-17 staining is very weak and very difficult to interpret. Following PMA/Ion restimulation, cells to be detected were surface labeled with CD4-APC and then stained with IL-17-PE for flow cytometric analysis. It has been a very sophisticated experimental method [11]. We used PMA/Ion restimulation just for two points. First of all, in this study, it has been confirmed that DCs maturation increased (the expression levels of CD40, CD80, CD86, and MHC-II were higher) after being stimulated by OVA. That means that antigen-specific response is higher through OVA stimulation (Table 1 and Figure 6). What is more, not only to detect antigen-specific response, our research is also focused on exploring the effect of DCs from MTX and CTX treated mice on specific T cell subsets, Th17 and Treg, when DCs antigen presentation reaction changes. Therefore, we need to use PMA/Ion restimulation and IL-17 and FoxP3 staining. 

In addition to DCs, MTX and CTX could affect the maturation of other antigen-presenting cells; for example, macrophages play an important role in RA pathogenesis. It has been reported that immunosuppressant drugs, such as MTX and CTX, induce macrophage apoptosis *in vitro* [39] or reduce macrophage infiltration [40]. As a result, it inhibits cytokine production and leukocyte migration to inflammatory foci [40]. Bulgarelli et al. [41] found that MTX led to downregulation of pro-inflammatory genes, such as TNF-*α*, IL-1*β*, and TLR2, and upregulation of the anti-inflammatory TGF-*β*1 gene. In this study, we mainly investigated the combinatorial effect of MTX and CTX on dendritic cells, the most important member in antigen presenting cells. It is that the cytokines produced by DCs and the signaling pathways of DCs regulating the balance of Th17/Treg cells, such as TLRs/MyD88 pathways, are our research priorities in the further research.

 In summary, our present study demonstrated that dendritic cells maturation is relevant to many factors such as the disease activity and drug action. Then it is closely related to the differentiation of Th17, Treg cells. MTX combined with CTX induced Treg skewing and Th17 suppression by attenuating maturation of DCs. Thus, it reduces antigen-driven T cell proliferation and prevents inflammation. It adds a novel mechanism and therapeutic target of MTX combined with CTX for autoimmune disease treatment.

## 5. Conclusions

The maturity of dendritic cells is consistent with the ability of stimulating the T cell proliferation. Th17 inflammatory response is stronger, while the level of DCs maturity is higher. In contrast, the immunosuppression of Treg is stronger. MTX combined with CTX significantly inhibits the DCs maturity and then induces Treg skewing and Th17 suppression, which tends to restore the balance of Th17/Treg. Our study adds a novel mechanism and therapeutic target of MTX combined with CTX for autoimmune disease treatment.

## Figures and Tables

**Table 1:** BMDC surface antigen's expression of OVA group compared with control.

Group	*n*	BMDC surface antigen's expression ($\bar{X}\pm S$, %)				
		CD11c	CD40	CD80	CD86	MHC-II
Control	6	82.57 ± 4.57	61.57 ± 3.50	81.90 ± 3.79	67.18 ± 3.21	70.30 ± 2.33
OVA Group	6	87.85 ± 4.51	69.72 ± 4.70*	89.78 ± 3.67*	79.37 ± 3.60*	84.32 ± 2.81*

Values are expressed as mean ± standard deviation; *t*-test was used for statistical analysis.

*P < 0.05 was considered as statistically significant.

**Table 2:** BMDC surface antigen's expression of OVA group.

Week	*n*	BMDC surface antigen's expression ($\bar{X}\pm S$, %)				
		CD11c	CD40	CD80	CD86	MHC-II
0	6	87.85 ± 4.51	69.72 ± 4.70	89.78 ± 3.67	79.37 ± 3.60	84.32 ± 2.81
3	5	88.50 ± 3.71	69.40 ± 3.82	89.10 ± 2.75	78.90 ± 4.63	83.82 ± 4.19
6	4	86.18 ± 4.25	68.25 ± 2.86	88.13 ± 3.18	77.88 ± 2.61	82.55 ± 3.02
9	5	85.60 ± 3.41	67.40 ± 3.56	87.20 ± 2.87	76.70 ± 2.97	81.50 ± 3.11

Values are expressed as mean ± standard deviation; the difference among groups was determined by ANOVA analysis and comparison between two groups was analyzed by the *t*-test.

No significant difference among different time of administration (*P* > 0.05).

**Table 3:** BMDC surface antigen's expression of MTX group.

Week	*n*	BMDC surface antigen's expression ($\bar{X}\pm S$, %)				
		CD11c	CD40	CD80	CD86	MHC-II
0	6	87.85 ± 4.51	69.72 ± 4.70	89.78 ± 3.67	79.37 ± 3.60	84.32 ± 2.81
3	5	86.68 ± 4.43	68.52 ± 1.77	88.18 ± 1.18	78.54 ± 2.41	83.40 ± 1.92
6	5	85.74 ± 3.14	66.28 ± 3.08	86.06 ± 1.95*	76.26 ± 2.53	81.18 ± 2.23*
9	6	84.42 ± 5.56	63.65 ± 1.82^∗#^	83.62 ± 2.14^∗#^	73.37 ± 1.41^∗#^	78.17 ± 2.02^∗#△^

Values are expressed as mean ± standard deviation; the differences of BMDC surface molecules, CD40, CD80, and CD86, at different time were determined by Kruskal-Wallis *H* test.

*Compared with 0 week, *P* < 0.05; ^#^compared with 3rd week, *P* < 0.05; ^△^compared with 6th week, *P* < 0.05.

**Table 4:** BMDC surface antigen's expression of CTX group.

Week	*n*	BMDC surface antigen's expression ($\bar{X}\pm S$, %)				
		CD11c	CD40	CD80	CD86	MHC-II
0	6	87.85 ± 4.51	69.72 ± 4.70	89.78 ± 3.67	79.37 ± 3.60	84.32 ± 2.81
3	5	87.22 ± 1.61	67.96 ± 2.83	87.88 ± 3.53	78.34 ± 4.27	83.20 ± 3.42
6	5	84.66 ± 3.29	65.48 ± 2.39*	85.36 ± 4.21*	74.90 ± 3.13*	79.94 ± 4.00*
9	5	83.78 ± 1.46*	62.92 ± 2.08^∗#^	82.70 ± 1.66^∗#^	72.00 ± 2.45^∗#^	75.32 ± 2.09^∗#△^

Values are expressed as mean ± standard deviation; the differences of BMDC surface molecules, CD40, at different time were determined by Kruskal-Wallis *H* test.

*Compared with 0 week, *P* < 0.05; ^#^compared with 3rd week, *P* < 0.05; ^△^compared with 6th week, *P* < 0.05.

**Table 5:** BMDC surface antigen's expression of MTX combined with CTX group.

Week	*n*	BMDC surface antigen's expression ($\bar{X}\pm S$, %)				
		CD11c	CD40	CD80	CD86	MHC-II
0	6	87.85 ± 4.51	69.72 ± 4.70	89.78 ± 3.67	79.37 ± 3.60	84.32 ± 2.81
3	5	86.10 ± 2.85	67.70 ± 3.97	87.40 ± 3.29	77.90 ± 3.83	82.44 ± 4.53
6	5	84.20 ± 3.18	64.20 ± 3.30*	84.00 ± 2.73*	73.50 ± 4.74*	77.56 ± 4.85^∗#^
9	6	83.32 ± 1.81*	59.47 ± 2.27^∗#△^	79.17 ± 1.94^∗#△^	68.17 ± 2.07^∗#△^	71.87 ± 2.20^∗#△^

Values are expressed as mean ± standard deviation; the differences of BMDC surface molecules, CD40, at different time were determined by Kruskal-Wallis *H* test. The DC surface antigen's expression was significantly decreased on 9th week.

*Compared with 0 week, *P* < 0.05; ^#^compared with 3rd week, *P* < 0.05; ^△^compared with 6th week, *P* < 0.05.

**Table 6:** Comparison of DC surface antigen's expression in each group on 9th week.

Groups	*n*	BMDC surface antigen's expression ($\bar{X}\pm S$, %)				
		CD11c	CD40	CD80	CD86	MHC-II
OVA	5	85.60 ± 3.41	67.40 ± 3.56	87.20 ± 2.87	76.70 ± 2.97	81.50 ± 3.11
MTX	6	84.42 ± 5.56	63.65 ± 1.82*	83.62 ± 2.14*	73.37 ± 1.41*	78.17 ± 2.02*
CTX	5	83.78 ± 1.46	62.92 ± 2.08*	82.70 ± 1.66*	72.00 ± 2.45*	75.32 ± 2.09*
MTX and CTX	6	83.32 ± 1.81	59.47 ± 2.27^∗#△^	79.17 ± 1.94^∗#△^	68.17 ± 2.07^∗#△^	71.87 ± 2.20^∗#△^

Values are expressed as mean ± standard deviation; the difference among groups was determined by ANOVA analysis and comparison between two groups was analyzed by the *t*-test. The DC surface antigen's expression was significantly decreased in MTX combined with CTX group.

*Compared with 0 week, *P* < 0.05; ^#^compared with 3rd week, *P* < 0.05; ^△^compared with 6th week, *P* < 0.05.

**Table 7:** Stimulation index of mixed lymphocytes stimulated by BMDC on 9th week.

Groups	*n* (number of wells)	T-cells proliferation (cpm)		
		$\bar{X}\pm S$	*F*	*P*
T-cell Control	9	17169.86 ± 2307.08^△▲^		
OVA	9	28734.21 ± 1091.63^∗△▲^		
MTX	9	23318.43 ± 528.13^∗#^	38.45	<0.001
CTX	9	20484.83 ± 2472.63^∗#^		
MTX and CTX	9	16043.62 ± 1721.36^#△▲^		

Values are expressed as mean ± standard deviation; the difference among groups was determined by ANOVA analysis and comparison between two groups was analyzed by the *t*-test.

*Compared with T cell Control, *P* < 0.05; ^#^compared with OVA group, *P* < 0.05; ^△^compared with MTX group, *P* < 0.05; ^▲^compared with CTX group, *P* < 0.05.
